# O-Sialoglycoprotein Endopeptidase Deficiency Impairs Proteostasis and Induces Autophagy in Human Embryonic Stem Cells

**DOI:** 10.3390/ijms25147889

**Published:** 2024-07-18

**Authors:** Hua Teng, Siyi Chen, Fang Liu, Yanling Teng, Yunlong Li, Desheng Liang, Lingqian Wu, Zhuo Li

**Affiliations:** Center for Medical Genetics, Hunan Key Laboratory of Medical Genetics, School of Life Sciences, Central South University, 110 Xiangya Road, Changsha 410078, China; ivy.tenghua@gmail.com (H.T.); chensiyi@sklmg.edu.cn (S.C.); liufang@sklmg.edu.cn (F.L.); tengyanling@sklmg.edu.cn (Y.T.); liyunlong@sklmg.edu.cn (Y.L.); liangdesheng@sklmg.edu.cn (D.L.)

**Keywords:** *OSGEP*, hESCs, proteostasis, tRNA modification

## Abstract

The *OSGEP* gene encodes O-sialoglycoprotein endopeptidase, a catalytic unit of the highly conserved KEOPS complex (Kinase, Endopeptidase, and Other Proteins of small Size) that regulates the second biosynthetic step in the formation of N-6-threonylcarbamoyladenosine (t6A). Mutations in KEOPS cause Galloway–Mowat syndrome (GAMOS), whose cellular function in mammals and underlying molecular mechanisms are not well understood. In this study, we utilized lentivirus-mediated *OSGEP* knockdown to generate *OSGEP*-deficient human embryonic stem cells (hESCs). *OSGEP*-knockdown hESCs exhibited reduced stemness factor expression and G2/M phase arrest, indicating a potential role of *OSGEP* in the regulation of hESC fate. Additionally, *OSGEP* silencing led to enhanced protein synthesis and increased aggregation of proteins, which further induced inappropriate autophagy, as evidenced by the altered expression of P62 and the conversion of LC3-I to LC3-II. The above findings shed light on the potential involvement of *OSGEP* in regulating pluripotency and differentiation in hESCs while simultaneously highlighting its crucial role in maintaining proteostasis and autophagy, which may have implications for human disease.

## 1. Introduction

Galloway–Mowat syndrome (GAMOS) is an extremely rare autosomal or X-linked recessive disorder that primarily affects the kidneys and central nervous system [1]. The incidence of GAMOS is less than one in a million [2]. At present, eleven causative genes have been identified: five KEOPS (Kinase, Endopeptidase, and Other Proteins of small Size) complex related-genes (*OSGEP*, *TP53RK*, *TPRKB*, *LAGE3*, and *GON7*) and six other genes (*WDR73*, *WDR4*, *NUP133*, *NUP107*, *YRDC*, and *PRDM15*) [3,4,5,6,7,8].

*OSGEP* (O-sialoglycoprotein endopeptidase) was first reported to be associated with GAMOS in 2017 [9]. To date, *OSGEP* variants in 45 patients from 38 families have been reported and are the most prevalent certain stemness markers of the genetic etiology of GAMOS [2,9,10,11,12,13,14,15,16]. Most affected individuals die in early childhood, and the primary mutation type of *OSGEP* is a missense mutation. In a previous study, the knockout of *OSGEP* in zebrafish and mouse models resulted in early lethality [9]. Such results suggest that the complete absence of *OSGEP* leads to cell death and that *OSGEP* is essential for normal growth and development in a variety of model organisms. However, research on its impact on humans is limited. Further investigation into the role of *OSGEP* in human cells is conducive to advancing our understanding of GAMOS. 

*OSGEP* as a catalytic unit of the KEOPS complex participates in the biosynthesis of N6-threonylcarbamoyl adenosine (t6A), a universal transfer RNA (tRNA) modification at position 37 that decodes ANN codons in eukaryotes and archaea [17]. tRNA modification is crucial for protein synthesis and is extensively characterized in *Saccharomyces cerevisiae*. The authors of several studies have linked tRNA hypomodification to elevated missense error rates and disrupted codon decoding efficiency, resulting in protein aggregation and harmful effects on the cell [18,19,20]. A number of studies have demonstrated the role of proteostasis in mouse neural stem cells (NSCs) and their ability to exit quiescence, self-renew, and differentiate [21,22]. To remove abnormal protein aggregates and maintain protein homeostasis, autophagy is employed. Autophagy plays a crucial role in maintaining neuronal homeostasis and synaptic function. In numerous neurodegenerative diseases marked by the accumulation of protein aggregates—such as Alzheimer’s disease, Parkinson’s disease, Huntington’s disease, and amyotrophic lateral sclerosis (ALS)—this process is often dysregulated [23,24,25,26]. *ELP3* (Elongator Acetyltransferase Complex Subunit 2) is a susceptibility gene for ALS and affects the lifespan of some ALS patients [27,28]. *ELP3* is involved in the modification of the wobble uridine (U34) of tRNA, and its mutants can lead to inappropriate autophagy [29]. The above studies suggest that tRNA modifications are closely related to protein homeostasis and autophagy. However, no study has elucidated the impact of the KEOPS complex on proteostasis and autophagy, especially in human cells. 

In the present study, we developed an *OSGEP*-knockdown hESC model by using lentivirus shRNA infection. Based on the hECS model, we detected the proliferation, apoptosis, and translation of stem cell markers to investigate the impact of *OSGEP* on hESC fate. Moreover, we examined protein synthesis rates, protein aggregation, and alterations in autophagy levels to explore the global protein changes during *OSGEP* deficiency. 

## 2. Results

### 2.1. OSGEP Knockdown Results in Reduced Proliferation and Increased Apoptosis

**Figure 1:**
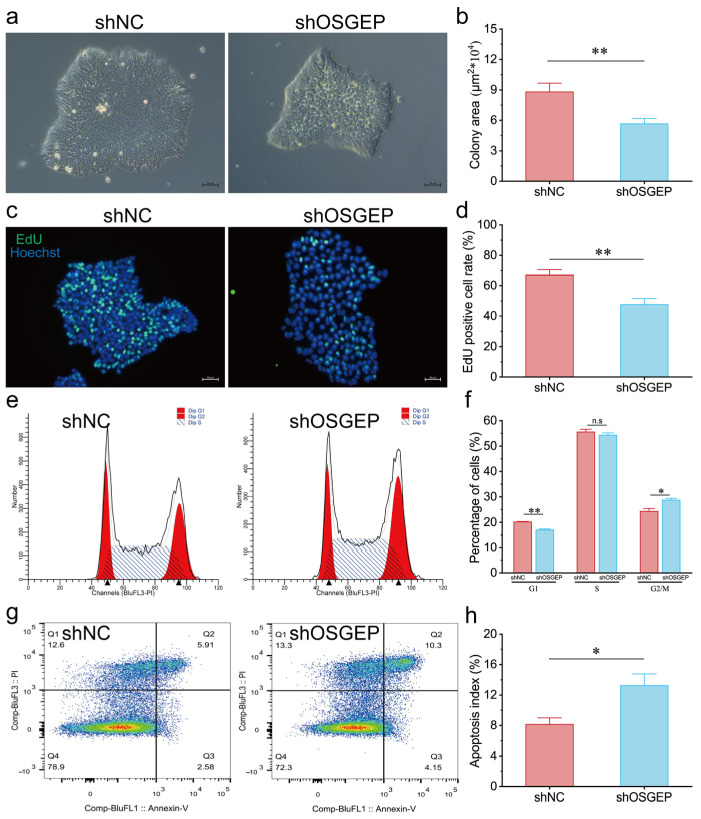
*OSGEP* knockdown affects hESC proliferation and apoptosis. shNC represents the control group, and sh*OSGEP* represents the *OSGEP*-knockdown group. (**a**) Images of the colony formation assay, scale bar: 50 μm; (**b**) quantification of the colony area in the colony formation assay; (**c**) images of EdU staining, scale bar: 50 μm; (**d**) quantification of the EdU-positive cell area; (**e**) the flow cytometry results using propidium iodide (PI) staining; (**f**) quantification of the cell cycle analysis; (**g**) the flow cytometry results of PI and Annexin-V; (**h**) quantification of the cell apoptosis index. n.s ≥ 0.05; * *p* < 0.05; ** *p* < 0.01.

To investigate the functional role of *OSGEP* in stem cells, we constructed *OSGEP*-knockdown (sh*OSGEP*) hESCs, which demonstrated reductions of 40% and 63% in *OSGEP* mRNA and protein levels, respectively (Figure S1). Our morphological analysis results revealed that *OSGEP* silencing maintained the typical dome-shaped colonies but with a reduced surface area and increased intercellular spacing (Figure 1a,b). The sh*OSGEP* hESCs showed a significantly reduced EdU staining area, indicating decreased cell proliferation (Figure 1c,d). Furthermore, *OSGEP* knockdown led to an enlargement of the nuclei compared with the control (Figure S2). Our cell cycle analysis results indicated a significant increase in the proportion of cells in the G2/M phase and a decrease in the proportion of cells in the G0/G1 phase in sh*OSGEP* hESCs (Figure 1e,f), suggesting *OSGEP* knockdown-induced cell cycle arrest in the G2/M phase. Additionally, our apoptosis analysis results revealed a higher cell apoptosis index in sh*OSGEP* hESCs (Figure 1g,h). The above findings collectively highlight the relevance of *OSGEP* in hESC proliferation and apoptosis.

### 2.2. OSGEP Knockdown Affects the Expression of Stemness Markers 

**Figure 2:**
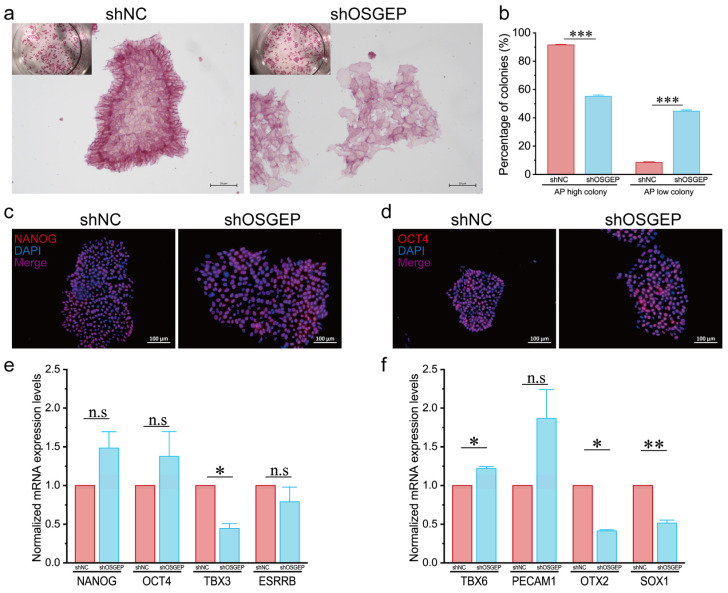
*OSGEP* knockdown affects expression of stemness markers. (**a**) Images of alkaline phosphatase (AP) staining, scale bar: 20 μm; (**b**) quantification of the percentage of high-AP and low-AP colonies; (**c**) representative images of NANOG staining; (**d**) images of OCT4 staining; (**e**) quantification of the mRNA expression of *NANOG*, *OCT4*, *TBX3*, and *ESRRB*; (**f**) quantification of the mRNA expression of *TBX6*, *PECAM1*, *OTX2*, and *SOX1*. n.s ≥ 0.05; * *p* < 0.05; ** *p* < 0.01; *** *p* < 0.001.

We performed alkaline phosphatase (AP) staining assays, in which high AP levels serve as a marker for undifferentiated states. Remarkably, we observed a significant decrease in the AP-positive population in sh*OSGEP* hESCs (Figure 2a,b). Additionally, we investigated several genes essential for maintaining the pluripotency of hESCs, including *NANOG*, *OCT4*, *TBX3*, and *ESRRB*. Although *NANOG* and *OCT4* showed no significant changes in protein and mRNA levels, a significant reduction in *TBX3* expression was noted (Figure 2c–e). Subsequently, we explored how the reduction in *OSGEP* affects the ability of hESCs to differentiate into various cell lineages. The increased expression of *TBX6* in sh*OSGEP* hESCs suggests that the cells might differentiate toward a mesodermal lineage. Elevated levels of *PECAM1* suggest that the sh*OSGEP* hESCs might differentiate into endothelial cells. The reduction in both *OTX2* and *SOX1* suggests a decrease in neural differentiation potential (Figure 2f). The above results indicate that *OSGEP* knockdown affects the expression of pluripotency markers of hESCs, making them more prone to differentiation and causing a shift away from the neural lineage.

### 2.3. OSGEP Deficiency Perturbs Proteostasis

**Figure 3:**
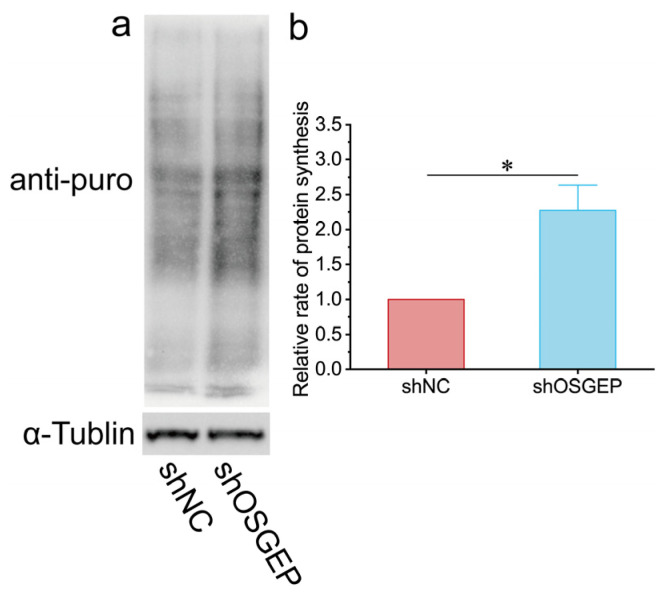
*OSGEP* knockdown affects protein synthesis rate. (**a**) An image of the puromycin incorporation assay and (**b**) the quantification of puromycin. * *p* < 0.05.

**Figure 4:**
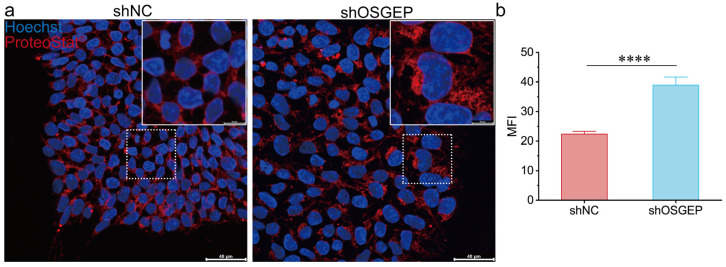
*OSGEP* knockdown leads to protein aggregation. (**a**) Images of the protein aggregation assay with immunofluorescence staining, scale bar: 40 μm and (**b**) the quantification of protein aggregation. MFI, mean fluorescence intensity. **** *p* < 0.0001.

Given the recognized influence of tRNA modifications on proteostasis, we examined the impact of *OSGEP* on global protein synthesis in hESCs. The results of a puromycin incorporation assay showed that de novo protein synthesis rates were remarkably elevated in sh*OSGEP* hESCs (Figure 3a,b). These findings were further validated in the HEK293T cell line (Figure S3). In sh*OSGEP* hESCs, numerous red punctate signals were observed in the cytoplasm (Figure 4a), and the fluorescence intensity was significantly higher than in the control cells (Figure 4b). We used MG-132 as a positive control, a relatively non-specific proteasome inhibitor known to accelerate the formation of perinuclear aggresomes and inclusion bodies. The MG-132 treatment group exhibited more numerous and stronger red fluorescent signals and fewer cells, indicating that the accumulation of proteins leads to cell death (Figure S4). The above results indicate that *OSGEP* depletion leads to elevated protein synthesis and the accumulation of aggregated proteins, highlighting the potential role of *OSGEP* in maintaining protein homeostasis in hESCs.

### 2.4. OSGEP Deficiency Induces Inappropriate Autophagy

**Figure 5:**
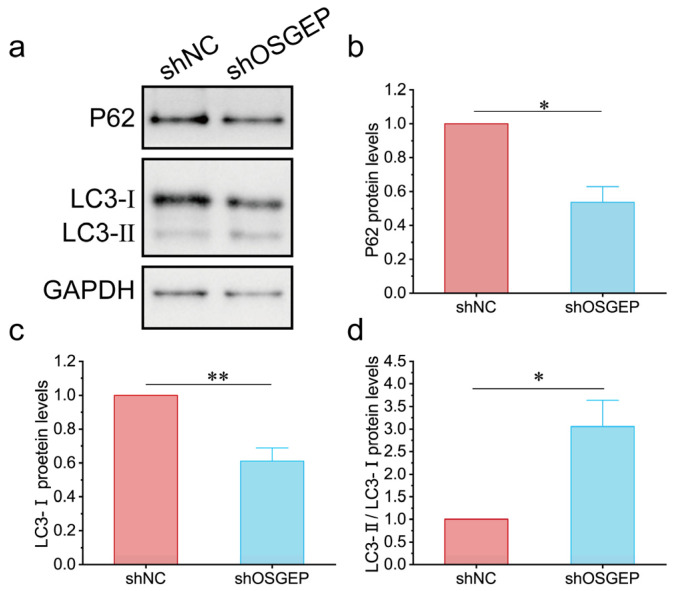
*OSGEP* deficiency induces inappropriate autophagy in hESCs. (**a**) An image of the Western blot of P62 and LC3; (**b**) quantification of the P62 protein expression level; (**c**) quantification of the LC3-I protein expression level; (**d**) quantification of the LC3-II/LC3-I protein expression level. Calibrated with GPAPH and normalized by the control group. * *p* < 0.05; ** *p* < 0.01.

To investigate the involvement of autophagy in aggregated proteins, we examined alterations in autophagy-associated protein levels. P62, a cargo protein specific to autophagosomes, forms aggregates that can be selectively degraded through autophagy [30]. The expression levels of P62 decreased in sh*OSGEP* hESCs (Figure 5a,b). The conversion of LC3-I to LC3-II serves as a key marker of autophagosome formation. Upon the silencing of *OSGEP*, we observed a notable decrease in LC3-I expression levels and a significant elevation in the LC3-II/LC3-I ratio (Figure 5c,d). The above findings collectively indicate that *OSGEP* depletion triggers autophagy as a cellular response to counteract the accumulation of aggregated proteins.

## 3. Discussion

In the study presented herein, we observed that *OSGEP* knockdown in hESCs led to decreased cell proliferation and increased apoptosis. These findings are consistent with observations in *OSGEP*-knockdown human podocyte cell lines [9], indicating that impaired cell proliferation and increased apoptosis are common pathogenic features of GAMOS.

Cell cycle regulation is important for the self-renewal and pluripotency of hESCs [31]. Our findings suggest that *OSGEP* silencing results in cell cycle arrest in hESCs, which may impact their self-renewal capacity. This finding is consistent with previous studies demonstrating that strongly mitotic tissues are highly sensitive to the loss of *kae1* (the *Drosophila* ortholog of *OSGEP*), whereas non-proliferating tissues are less affected [32]. Additionally, the results of a previous study showed that the KEOPS complex may inhibit Mis17-Mis6 complex-mediated centromere formation in *Schizosaccharomyces pombe* [33], which could be associated with G2/M phase arrest in hESCs. 

The results of our puromycin incorporation assay demonstrate that *OSGEP* knockdown leads to increased protein synthesis in both hESCs and HEK293T cells. The observed increase in protein synthesis may be related to an increase in the frequency of leaky scanning through start codons, leading to enhanced protein translation. This phenomenon has also been observed in yeast lacking the function of SUA5/YDRC, which is involved in t6A modifications [34]. Additionally, in yeast with *kae1* mutations, the upregulation of genes related to amino acid synthesis can be observed [35]. In our study, an abnormal increase in protein aggregates in *OSGEP*-knockdown hESCs was identified. This observation supports the result of increased protein synthesis, potentially due to erroneous translation products caused by defective t6A modifications. The results of previous studies have shown that abnormalities in t6A modifications can lead to increased +1 frameshift frequencies and readthrough of stop codons [34]. Whether the increase in abnormal protein synthesis observed in *OSGEP*-knockdown human cells is due to the same underlying mechanism requires further investigation.

In our study, we observed that *OSGEP* knockdown in hESCs leads to increased autophagic activity. This enhanced autophagy likely represents a cellular attempt to clear the increased protein aggregates resulting from *OSGEP* deficiency. Maintaining proper proteostasis is a fundamental requirement for cellular functionality and viability. Our results indicate that *OSGEP* knockdown affects protein synthesis, which in turn impacts the proliferation and apoptosis of hESCs, highlighting the importance of t6A modification in maintaining protein homeostasis.

In conclusion, the results of our study elucidate the impact of *OSGEP* on stem cell development, demonstrating that *OSGEP* is crucial for maintaining the fate of hESCs. Moreover, our study results reveal that *OSGEP* deficiency leads to proteostasis dysregulation and autophagy activation. The above findings thus provide new insights into the pathogenic mechanisms of GAMOS syndrome. Further research is warranted to unravel the intricate mechanisms underlying the interplay between *OSGEP*, proteostasis, and autophagy in the context of neurodevelopmental disorders.

## 4. Materials and Methods

### 4.1. Cell Culture

H9 hESCs were regularly cultured in mTeSR Plus medium (STEMCELL Technologies, Vancouver, BC, Canada) on plates coated with Matrigel. HEK293T cells were grown in DMEM supplemented with 10% fetal bovine serum (FBS). Cell passaging was performed at 70–80% confluence. All cell cultures were maintained in a humidified incubator set at 37 °C with 5% CO_2_.

### 4.2. Lentivirus Production and Transduction of hESCs

To generate *OSGEP*-knockdown hESCs, hESCs were infected with pLKO.1 lentiviruses containing OSGEP shRNA1 (sh1), OSGEP shRNA2 (sh2), and a non-target shRNA (negative control, NC). The sequences of shRNAs used are listed in Table S1. Briefly, HEK293T cells were transfected with the specified lentiviral vector utilizing Lipofectamine 3000 reagent (Invitrogen, Waltham, MA, USA) following the manufacturer’s guidelines. The target and packaging plasmids psPAX2 and pMD2.G (VSV-G envelope protein) were used in a 3:2:1 ratio. After 48 h, the culture medium was collected and filtered with a 0.45 μm filter (Millipore, Burlington, MA, USA). Subsequently, hESCs were incubated with the obtained lentivirus-containing medium at 37 °C for 12 h. Following an additional 24 h, cells were selected by using 1 μg/mL puromycin for 1 week. 

### 4.3. HESC Self-Renewal and Proliferation Analysis

For the colony formation assay, 1000 cells were plated in 6-well plates. Colony size was assessed on day 7. Alkaline phosphatase (AP) staining was carried out using the Alkaline Phosphatase Staining Kit for Stem Cell (Maokang Biotechnology Co., Ltd., Shanghai, China). Briefly, 2–3 days after seeding, cells were washed with PBS, fixed for 2–5 min, and then incubated with AP solution for 5–10 min at 25 °C, shielded from light. Stained colonies were examined and documented using a microscope. At least 100 colonies per sample were counted for data analysis.

### 4.4. Flow Cytometry Analysis

For cell cycle analysis, cells were collected using Accutase (STEMCELL Technologies) and fixed overnight in 70% ice-cold ethanol. After fixation, cells were stained for 30 min with a solution containing 100 μg/mL RNase A and 50 μg/mL propidium iodide (PI) (Coolaber, Beijing, China). Flow cytometry was performed with a DxP Athena™ flow cytometer, and the data were analyzed using ModFit software version 4.1. To measure apoptotic cells, the Annexin V-FITC/PI Apoptosis Detection Kit (Vazyme, Nanjing, China) was used according to the manufacturer’s instructions. A flow cytometer and FlowJo version 10.8.1 were used for the analysis of the flow cytometric results. For the assessment of protein aggregation, cells were stained by using the Proteostat Protein Aggregation Assay Kit (Enzo Life Science, Farmingdale, NY, USA) following the manufacturer’s guidelines. 

### 4.5. Immunofluorescence Staining

Cells were seeded onto Matrigel-covered glass coverslips in mTeSR Plus medium. The cells were fixed in 4% paraformaldehyde for 20 min and incubated at 4 °C in a blocking buffer (PBS containing 5% BSA). The cells were stained in accordance with the manufacturer’s procedure and then washed three times in PBS. 

### 4.6. Real-Time Quantitative PCR

Total RNA was extracted using TRIzol reagent (Invitrogen). For cDNA synthesis, 2 μg of RNA was utilized with the RevertAid First Strand cDNA Synthesis Kit (Thermo Scientific, Waltham, MA, USA). Gene expression analysis was performed using the PowerUp SYBR Green Master Mix (Applied Biosystems, Waltham, MA, USA) on an ASA-9600 RT-qPCR System (BAIYUAN GENE-TECH, Lanzhou, China). Relative gene expression was determined using the comparative cycle threshold (Ct) method and analyzed using the 2^−ΔΔCt^ method. The RT-qPCR primer details are provided in Table S2. 

### 4.7. Measurement of Protein Synthesis by Puromycin Incorporation

The cells were incubated for 30 min in complete culture media containing 10 μg/mL puromycin. After treatment, the cells were washed twice with PBS, collected, and lysed using loading buffer. We used an anti-puromycin antibody to detect the puromycin by Western blot.

### 4.8. Western Blot Assay

Cell pellets were collected and lysed with a loading buffer. The lysates were denatured by heating at 100 °C for 10 min. Proteins from the whole-cell lysates were separated using SDS-PAGE and transferred to PVDF membranes (Millipore). The membranes were then incubated with specific primary antibodies, as detailed in Table S3, and the antibody–protein complexes were visualized using secondary antibodies conjugated to horseradish peroxidase (Sigma, Tokyo, Japan) and the SuperSignal™ West Pico PLUS Chemiluminescent Substrate (Thermo Scientific). The images were captured by the Molecular Imager ChemiDoc XRS+ System. 

### 4.9. Quantification and Statistical Analysis

We used ImageJ 1.53a to quantify the fluorescence intensity values and Western blot (WB) grayscale values, and we used Origin 2022 for data visualization. All results are representative of at least three independent experiments. The data are presented as the mean + SEM (Standard Error of the Mean). We used a *t*-test to perform the significance analysis. The *p*-value levels are denoted as follows: n.s ≥ 0.05; * < 0.05; ** < 0.01; *** < 0.001; **** < 0.0001.

## Data Availability

The raw data supporting the conclusions of this article will be made available by the authors on request.

## Supplementary Materials

The supporting information can be downloaded at: https://www.mdpi.com/article/10.3390/ijms25147889/s1.
