# Supplementary material for: O-Sialoglycoprotein Endopeptidase Deficiency Impairs Proteostasis and Induces Autophagy in Human Embryonic Stem Cells
Source: Int J Mol Sci. 2024 Jul 18;25(14):7889. doi: 10.3390/ijms25147889 (PMC11277037; doi:10.3390/ijms25147889)
Supplement: Supplementary file 1 [file ijms-25-07889-s001.zip › ijms-3067168-supplementary/IJMS-Supplementary.pdf]

Figure S1

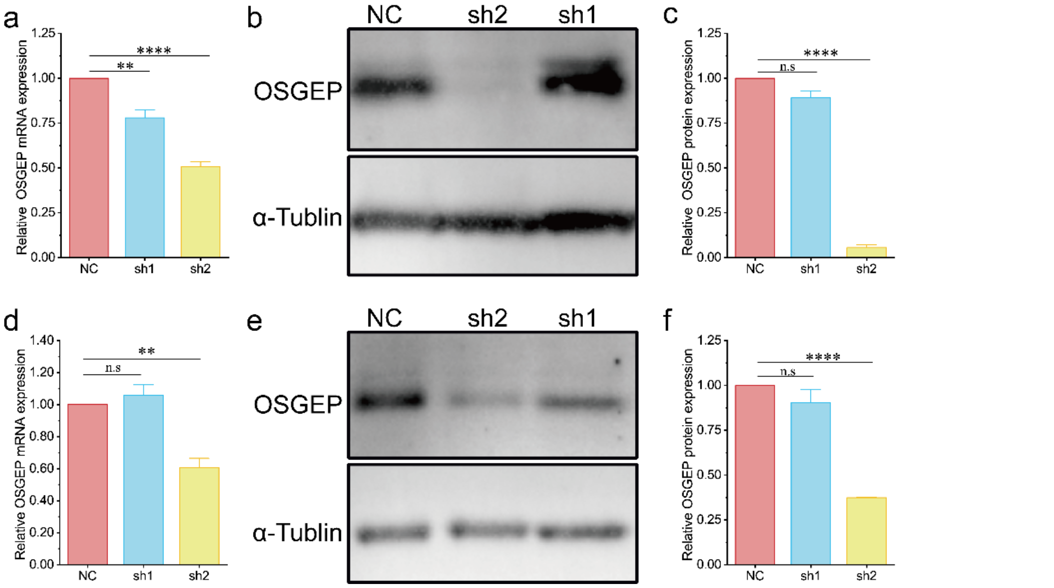

Figure S1 Generation of OSGEP knockdown cell lines. Cells were infected with pLKO.1 lentiviruses con-taining OSGEP shRNA1 (sh1), OSGEP shRNA2 (sh2), and a non-target shRNA (negative control, NC). a-c. OSGEP knockdown efficiency in HEK293T cells. a. RT-qPCR analysis of OSGEP mRNA expression. b. The image of Western Blot of OSGEP. c. The quantification of OSGEP protein ex-pression. d-f. OSGEP knockdown efficiency in hESCs. d. RT-qPCR analysis of OSGEP mRNA expression. e. The image of Western Blot of OSGEP. f. The quantification of OSGEP protein ex-pression.

Figure S2

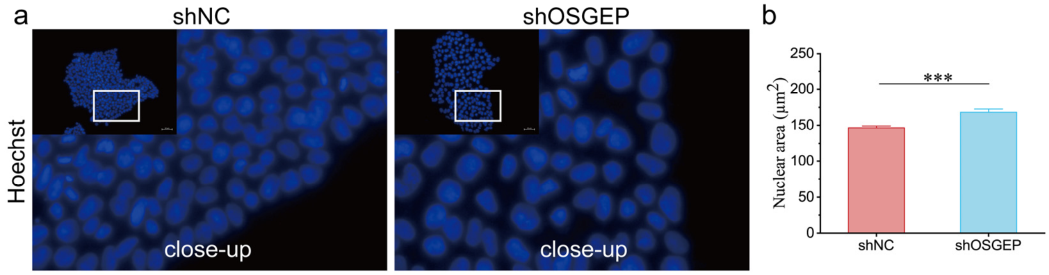

Figure S2 OSGEP knockdown led to an enlargement of the nuclei. a.The images of nuclear staining in EdU assay. b. Qquantification of nuclear area in EdU assay.

Figure S3

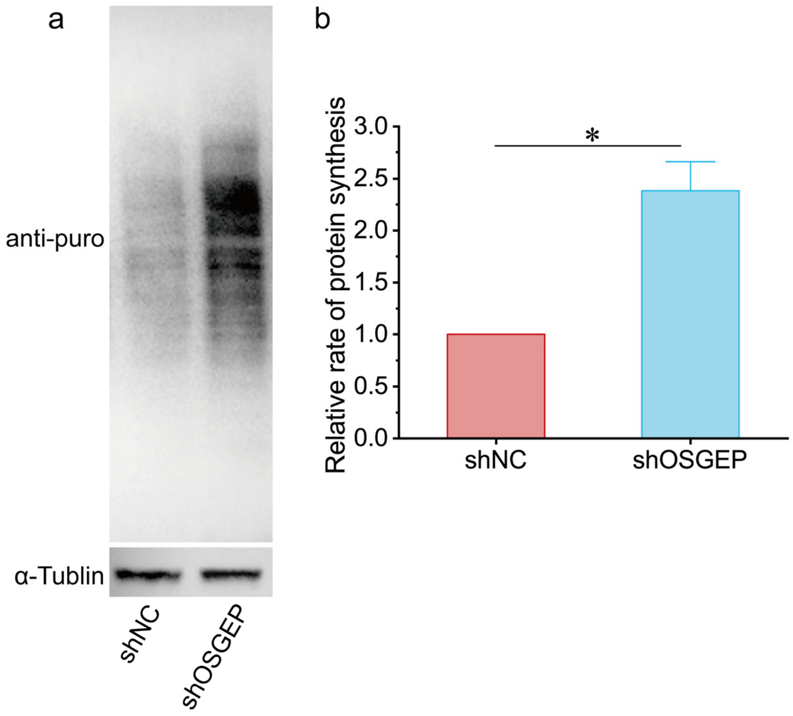

Figure S3 OSGEP knockdown increased protein synthesis in HEK293T cells. a. The images of puromycin incorporation assay in HEK293T cells. b. Quantification of puromycin.

Figure S4

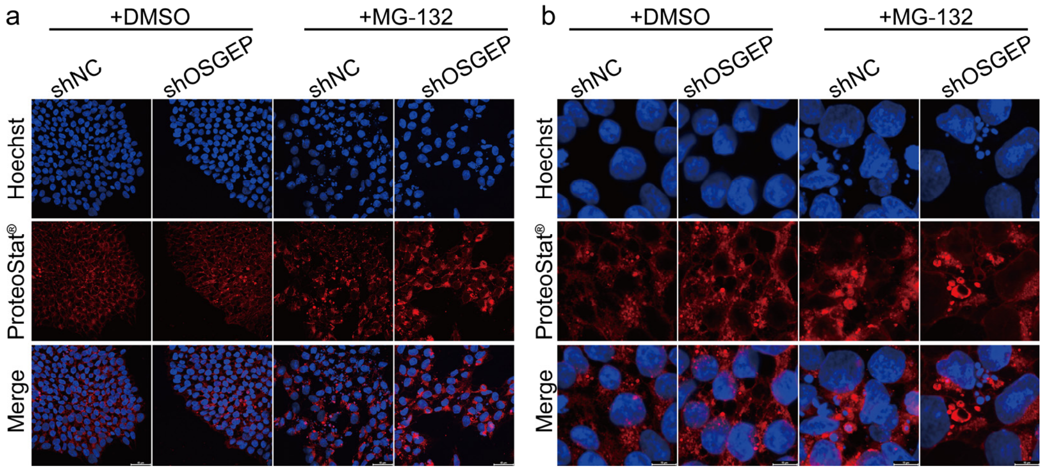

Figure S4 Protein aggregation assay of control and OSGEP-knockdown hESCs. a. The images of protein aggregation detected by immunofluorescence staining. b. 5 times the magnification of (a). MG-132, positive control. DMSO, solvent control.

Table S1. List of shRNA target sequences used in this study

Gene		shRNA target sequence
OSGEP	shRNA1 (sh1)	GGTGTAATGTGAGGCTACAGGAGAT
(NM_017807.3)	shRNA2 (sh2)	GGATTAACCTCCCAGGATATC

Table S2. List of primary antibodies used

Target antigen	Vendor	Dilution
OSGEP	Novus Biologicals	1:500
α tubulin	Cell Signaling Technology	1:5000
Lamin B1	Proteintech	1:10000
puromycin	Kerafast	1:2000
P62	Proteintech	1:4000
LC3	Proteintech	1:2500
GAPDH	Abcam	1:5000

Table S3. Sequences of the primers used in this study

Gene	Primer Sequence (5'-3')
OSGEP	F: CAGGAGGCACTAACAGAGTCT<br>R: ACCCACCAATGGCTTATTCCA
TBP	F: CCACTCACAGACTCTCACAAC<br>R: CTGCGGTACAATCCCAGAACT
OTX2	F: CAAAGTGAGACCTGCCAAAAAGA<br>R: TGGACAAGGGATCTGACAGTG
SOX1	F: CAGTACAGCCCCATCTCCAAC<br>R: GCGGGCAAGTACATGCTGA
TBX6	F: CATCCACGAGAATTGTACCCG<br>R: AGCAATCCAGTTTAGGGGTGT
PECAM1	F: AACAGTGTTGACATGAAGAGCC<br>R: TGTAAAACAGCACGTCATCCTT
NANOG	F: AAGGTCCCGGTCAAGAAACAG<br>R: CTTCTGCGTCACACCATTGC
OCT4	F: GAGAACCGAGTGAGAGGCAACC<br>R: CATAGTCGCTGCTTGATCGCTTG
TBX3	F: GAGGCTAAAGAACTTTGGGATCA<br>R: CATTTCGGGGTCGGCCTTA
ESRRB	F: ATCAAGTGCGAGTACATGCTC<br>R: CGCCTCCGTTTGGTGATCTC
