# Supplementary figures and images for: O-Sialoglycoprotein Endopeptidase Deficiency Impairs Proteostasis and Induces Autophagy in Human Embryonic Stem Cells
Source: Int J Mol Sci. 2024 Jul 18;25(14):7889. doi: 10.3390/ijms25147889 (PMC11277037; doi:10.3390/ijms25147889)

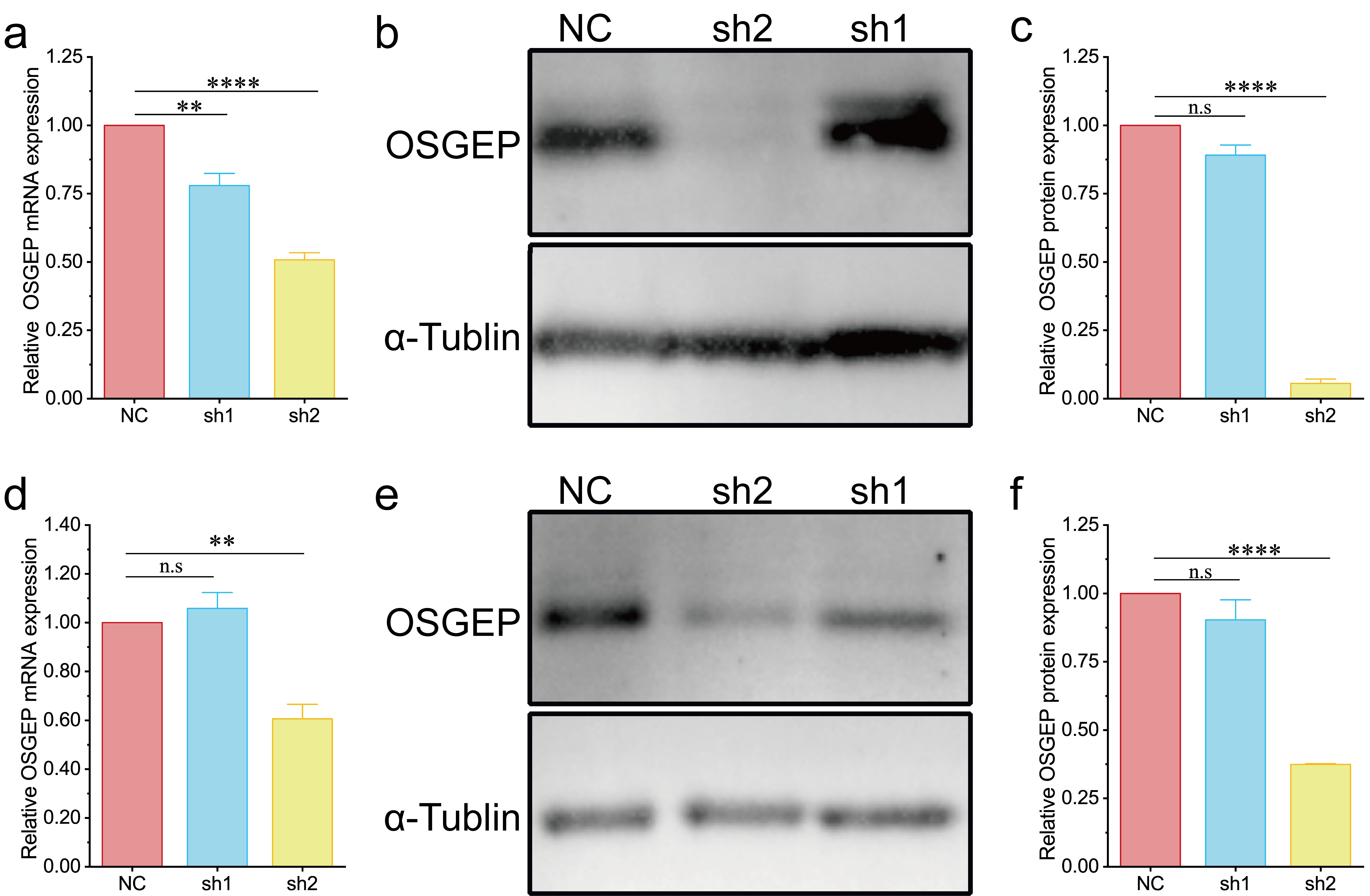

Supplement: Supplementary file 1 [file ijms-25-07889-s001.zip › ijms-3067168-supplementary/Figure S1.tif]

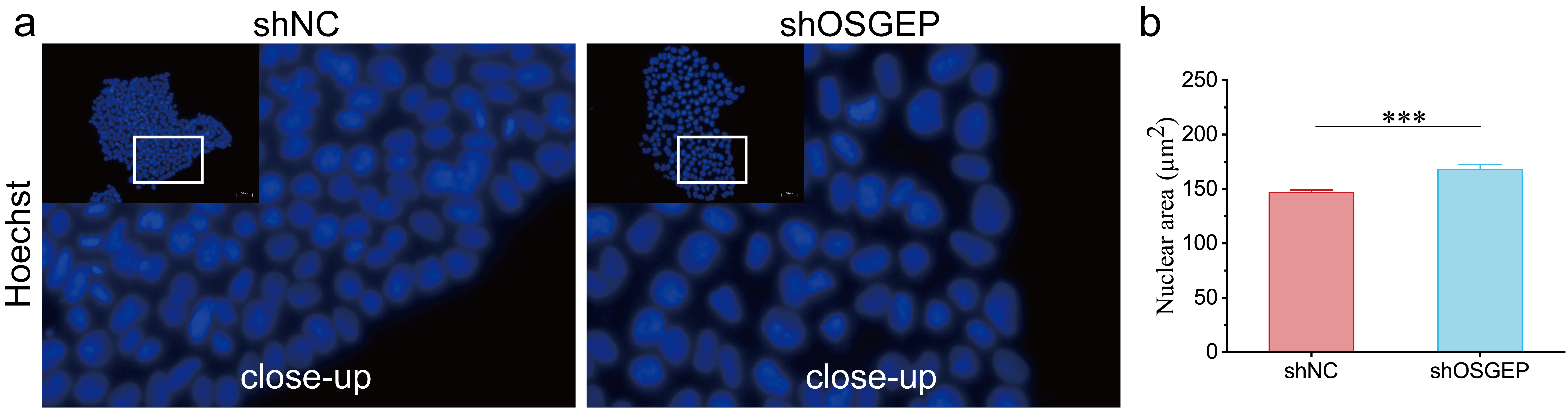

Supplement: Supplementary file 1 [file ijms-25-07889-s001.zip › ijms-3067168-supplementary/Figure S2.tif]

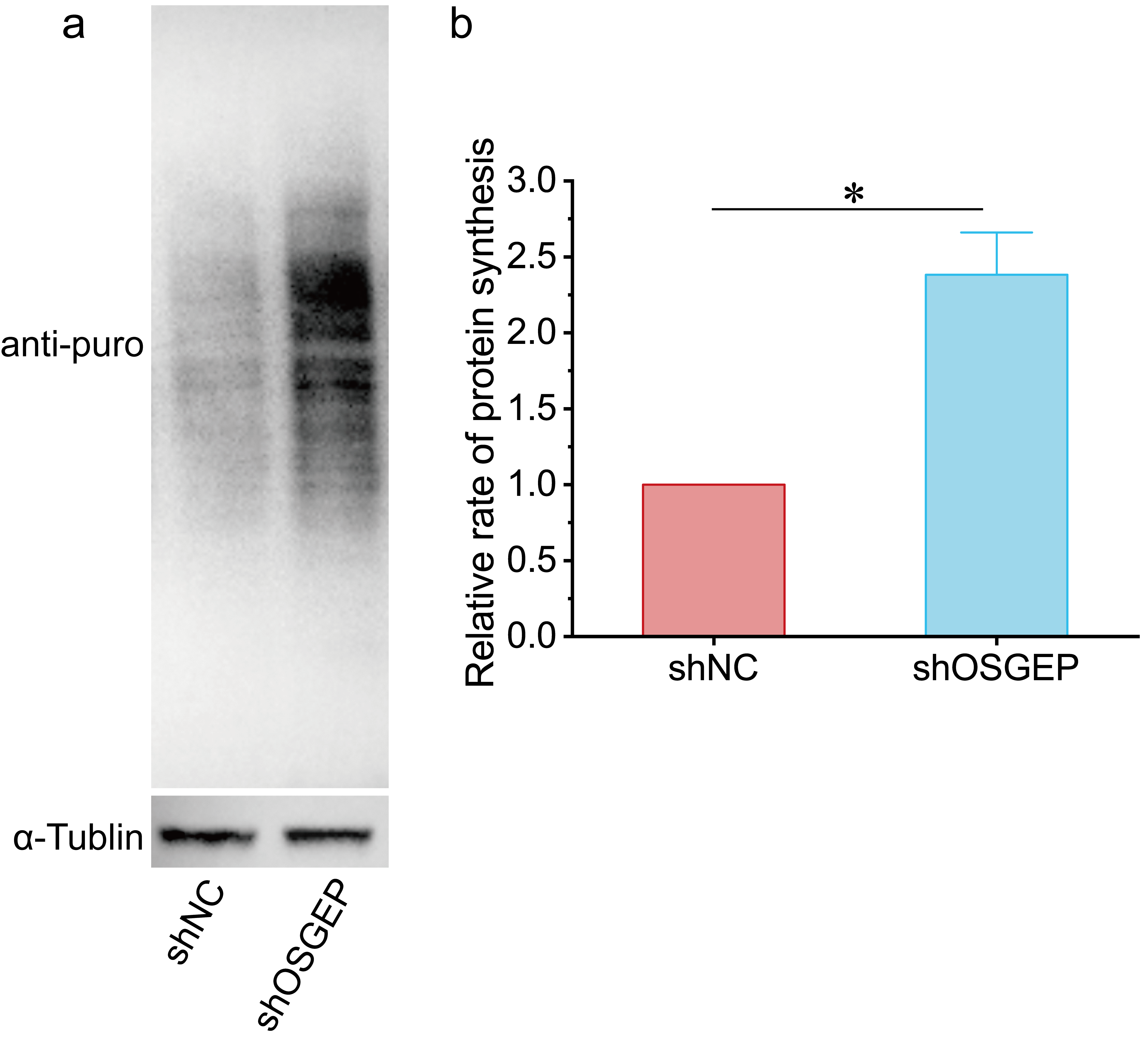

Supplement: Supplementary file 1 [file ijms-25-07889-s001.zip › ijms-3067168-supplementary/Figure S3.tif]
